# Evaluation of *Staphylococcus aureus* Nasal Carriage Screening before Vascular Surgery

**DOI:** 10.1371/journal.pone.0038127

**Published:** 2012-06-07

**Authors:** Jeroen M. W. Donker, Lijckle van der Laan, Yvonne J. A. M. Hendriks, Jan A. J. W. Kluytmans

**Affiliations:** 1 Department of Surgery, Amphia Hospital, Breda, The Netherlands; 2 Department of Microbiology and Infection Prevention, Amphia Hospital, Breda, The Netherlands; 3 Department of Microbiology and Infection Prevention, VUmc, Amsterdam, The Netherlands; National Institutes of Health, United States of America

## Abstract

**Introduction:**

Staphylococcus aureus is the most important pathogen in the development of surgical site infections (SSI). Patients who carry *S. aureus* in the nose are at increased risk for the development of SSI in cardiothoracic and orthopedic surgery. In these populations it has been shown that the risk for SSI can be substantially reduced by eradicating *S. aureus* carriage. For vascular surgery the relation between nasal carriage and surgical site infections has not been clearly investigated. For this reason we performed this study to analyze the relation between *S. aureus* nasal carriage and SSI in our vascular surgery population.

**Methods:**

A prospective cohort study was undertaken, including all patients undergoing vascular surgery between January first 2010 and December 31th 2010. Before surgery patients were screened for *S. aureus* nasal carriage using a PCR technique. The presence of SSI was recorded based on criteria of the CDC.

**Results:**

Screening was performed in 224. Of those, 55 (24.5%) were positive, 159 (71.0%) were negative and 10 (4.5%) were inconclusive. In the screened vascular population 4 *S. aureus* SSI occurred in the 55 carriers compared with 6 in 159 non-carriers (p = 0.24). A stratified analysis revealed a 10-fold increased risk in nasal carriers undergoing central reconstruction surgery (3 *S. aureus* SSI in 20 procedures versus 1 in 65 procedures in non-carriers, p = 0.039).

**Discussion:**

In patients undergoing central reconstruction surgery nasals carriers are at increased risk for the development of *S. aureus* SSI. These patients will probably benefit from perioperative treatment to eradicate nasal carriage.

## Introduction

*Staphylococcus aureus* nasal carriage increases a patient’s risk for developing a health care-associated infection with this micro organism, at least after cardiac surgery, orthopedic surgery and in peritoneal dialysis. [1], [2], [3], [4], [5] Preoperative screening for nasal carriage and subsequent treatment of carriers with mupirocin and chlorhexidine reduces the risk for the development of hospital-acquired *S. aureus* infections by 79% for deep-seated infections and 55% for superficial infections. [6] Consequently, the mean duration of hospital stay is reduced in treated carriers by approximately 2 days. A cost benefit analysis shows that the strategy is cost-effective and saves lives. [7].

In vascular surgery little is known about the relation between nasal carriage of *S. aureus* and surgical site infections (SSI).

For this reason we conducted a prospective analysis of *S. aureus* nasal carriage in patients undergoing vascular surgery and the occurrence of surgical site infections.

## Methods

A prospective cohort study was performed on all patients who underwent elective vascular surgery between January 1^st^ 2010 and December 31th 2010 in the Amphia Hospital, Breda, The Netherlands.

Operations included were; central reconstructions for both abdominal aortic aneurysms and occlusive disease (endovascular (EVAR) and open), peripheral bypass procedures (autologous and PTFE), endarterectomies of the femoral and carotid artery, embolectomies and Artero-venous access procedures.

Patients were screened on the day that they were admitted to the vascular surgery department of the Amphia hospital in Breda. Screening was performed using a dry, sterile swab, which was rotated four times in each nostril. The swab was placed in saline and centrifuged. Part of the sample was processed for polymerase chain reaction (PCR) on the presence of *S. aureus*, and part was inoculated onto a blood agar plate, to allow nasal and infecting strains to be compared in case a surgical site infection did occur.

The GeneXpert MRSA/SA Assay (Cepheid, Sunnyvale, CA) is a real-time PCR-based method, which identifies *S. aureus* and also can differentiate whether a *S. aureus* is a Methicillin-susceptible (MSSA) or Methicillin-resistant (MRSA). [8], [9], [10], [11].

Patients were followed prospectively for the development of Surgical site infections (SSI) which were defined according to the criteria of the Centers for Disease Control. [12].

**Table 1:** Baseline and surgical characteristics and surgical site infections caused by *S. aureus*.

Characteristics				
N	224			
Sex, Male/Female (%)	171/53 (76.3/23.7)			
Age mean (SD)	70 (10.1)			
Type of Surgery N/(%)				
			N° of SSI’s (%)	N° of *S. aureus* SSI (%)
Aortic open repair	52 (23)		6 (11)	3 (6)
Aortic endovascular repair	38 (17)		1 (3)	1 (3)
Femoral endarterectomy	34 (15)		3 (9)	3 (9)
Peripheral bypass surgery				
Autologous bypass	42 (66)		2 (5)	1 (2)
PTFE bypass	22 (34)		4 (18)	1 (5)
AV access surgery	14 (6)		0 (0)	0 (0)
Peripheral embolectomy	4 (2)		1 (25)	1 (25)
Carotid endarterectomy	18 (8)		0 (0)	0 (0)
Total	224 (100)		17 (8)	10 (4)

The main criteria are the presence of: redness, heat, swelling or pain around the wound within 30 days after the initial procedure, and the presence of a positive culture, drainage of the wound, or pus after a diagnostic puncture. When prosthetic material had been used the follow up was extended up to one year. Infections were differentiated between superficial, deep seated and organ based infections.

Screening was performed as part of the infection control strategy of the Amphia hospital using non-invasive sampling. Approval of the medical ethical committee and informed consent were not applicable.

**Table 2:** Relation between *S. aureus* carriage and surgical site infections caused by *S. aureus*.

Surgery type									
		*S. aureus* SSI-rate (%)		RR		95% CI		P-value*	
Central reconstructions (n = 90)									
Non-carriers (n = 65)		1 (1,5)							
Carriers (n = 20)	3 (15)		9.8		1.1–88.6		0.039		
Inconclusive (n = 5)**		0							
Other procedures (n = 134)									
Non-carriers (n = 94)		5 (5)							
Carriers (n = 35)		1 (3)		0.5		0.1–4.4		0.48	
Inconclusive (n = 5)**		0							

* Fisher’s exact test;

** Inconclusive screening results were not used for analyzation.

A stratified analysis was performed for patients with central vascular surgery, as we expected a possible difference for the importance of nasal carriage between patients suffering from peripheral arterial occlusive disease (PAOD) and patients suffering from central diluting vascular disease.

Patients suffering from PAOD have a gradient of lower limb ischemia, which ranges from impaired walking distance, due to inappropriate blood flow to the lower limbs (intermittent claudication), to critical limb ischemia. In those patients hypo perfusion of the lower limbs often results in ischemia or even ischemic ulcers. These ulcers may be colonized with pathogens, which may be introduced into the surgical wound. This may alter the role of nasal carriage as there is an additional source of *S. aureus* in the patient.

**Table 3:** Relation between *S. aureus* surgical site infections and risk factors for SSI.

Factor		Univariate			Multivariate	
	RR	95% CI	P-value	RR	95% CI	P-value***
Duration of the surgical procedure	NA		0.62**	1.0	0.98–1.02	0.89
						
ASA class 1 or 2 (compared to >2)	2.9	0.4–21.5	0.29*	3.4	0.4–30.2	0.26
						
BMI	NA		0.99**	1.0	0.88–1.34	0.89
						
*S. Aureus* screening	9.8	1.1–88.6	0.039*	12.8	1.1–147.9	0.041

* Fisher exact test;

** t-test;

*** Chi-square.

Statistical analyses was performed with SPSS software v 19.0 (SPSS Inc., Chicago, IL, USA), the Fisher exact test was used to determine significance.

A multivariate analysis was performed for evaluation of several other known risk factors on the development of SSI’s. Chi-square test was used to determine significance.

A *P-*value <0,05 was considered significant.

## Results

As shown in Table 1, 224 patients were included. There were a total of 17 SSI’s, 13 of which were superficial, and 4 where deep seated SSI’s. The PCR of nasal swabs showed that 159 (71.0%) were negative for *S. aureus*, 55 (24.6%) were positive and 10 (4.5%) were inconclusive because of inhibition of the amplification reaction. In 214 patients with conclusive results, there were 16 surgical site infections, 10 of which were caused by *S. aureus* and 6 by other pathogens.

The incidence of surgical site infections in nasal carriers of *S. aureus* is 4 out of 55 (7.3%), whereas the incidence in non-carriers is 6 out of 159 (3.8%)(RR = 1.9, 95%CI 0.5–7.5).

A stratified analysis was performed for central reconstruction surgery, peripheral bypass surgery and other procedures as shown in Table 2. In peripheral bypass surgery 2 *S. aureus* surgical site infections occurred in patients who did not carry *S. aureus* (n = 45) and no infections in Patients who carried *S. aureus* (n = 17) (*P>*0.05).

In the central reconstruction surgery population, there was 1 surgical site infection in patients who did not carry *S. aureus* (n = 65), this SSI occurred after an aortoilliac bypass procedure because of occlusive disease, and there were 3 infections with *S. aureus* in Patients who carried *S. aureus* (n = 20), 1 SSI after an EVAR procedure, 1 SSI after open aneurysm repair and 1 after an aortoilliac bypass procedure because of occlusive disease (RR = 9.8, 95%CI 1.1–88.6, P = 0.039).

A multivariate analysis including 3 other risk factors known from the literature for the development of surgical site infections, did not alter the effect of nasal carriage (Table 3).

## Discussion

Our study shows that surgical site infections in vascular surgery occur relatively frequent and that the majority (62%) are caused by *S. aureus*. Especially the central reconstructions and the peripheral bypass procedures have a relatively high incidence of surgical site infections, compared to, for example, carotid endartectomies and AV access procedures.

Overall there is no significant relation between nasal carriage of *S. aureus* and the occurrence of surgical site infections. However, a stratified analysis on patients who underwent abdominal aortic surgery shows a significant association. The effect in this group is comparable to what has been found previously in cardiothoracic and orthopedic surgery. [1], [2], [3], [5].

In other vascular procedures no significant effect was found. The infections in this group mainly occurred in peripheral procedures of patients with occlusive disease. Patients with occlusive vascular disease cope with insufficient blood flow to at least one of the, mostly lower, limbs. This insufficient blood flow is often associated with ischemic disease, e.g. gangrene of non healing ulcers. As this wounds can be infected or colonized prior to surgery with a large scale of different pathogens, this could limit the role of *S. aureus* nasal carriage. In our study no significant effect after peripheral vascular surgery was found. However, for one patient who was positive for *S. aureus* nasal carriage and who developed a *S. aureus* SSI, accidently a sample of both the nasal swab as well as a wound swab were available for typing. This showed that the two trains were identical. Considering the small number of patients and the frequent presence of wounds before surgery we consider the role of nasal carriage in peripheral vascular surgery unresolved.

All *S. aureus* strains were methicillin susceptible and no MRSA was found which reflects the low rate of MRSA in Dutch hospitals. Also all strains were mupirocin susceptible. Potentially administration of mupirocin could reduce the risk of nasal carriage. [6] A cost effectiveness analysis showed that treating every patient with *S. aureus* eradication therapy, without screening for nasal carriage is the most cost-effective way for preventing surgical site infections. [7] However, as recent studies reported mupirocin resistant MRSA strains [13], [14], it should only be used in proven MSSA and MRSA carriers to limit the risk for development of further resistance.

Based on the results of this study we conclude that *S. aureus* carriers who undergo central reconstructive surgery have a significant higher risk for the development of SSI which can be decreased by perioperative eradication of *S. aureus* in nasal carriers. [6].

This is important because infection with *S. aureus* after aortic reconstructions is related to severe complications and a high mortality rate.
